# Untapped Bounty: Sampling the Seas to Survey Microbial Biodiversity

**DOI:** 10.1371/journal.pbio.0050085

**Published:** 2007-03-13

**Authors:** Liza Gross

**Figure:**
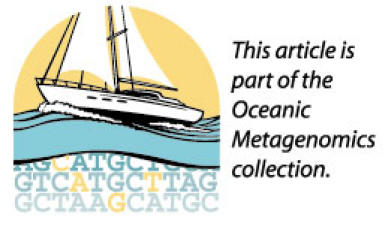


Being invisible to the naked eye, microbes managed to escape scientific scrutiny until the mid-17th century, when Leeuwenhoek invented the microscope. These cryptic organisms continued to thwart scientists' efforts to probe, describe, and classify them until about 40 years ago, owing largely to a limited morphology that defies traditional taxonomic methods and an enigmatic physiology that makes them notoriously difficult to cultivate.

Most of what we know about the biochemical diversity of microbes comes from the tiny fraction that submit to lab investigations. Not until scientists determined that they could use molecular sequences to identify species and determine their evolutionary heritage, or phylogeny, did it begin to become apparent just how diverse microbes are. We now know that microbes are the most widely distributed organisms on earth, having adapted to environments as diverse as boiling sulfur pits and the human gut. Accounting for half of the world's biomass, microbes provide essential ecosystem services by cycling the mineral nutrients that support life on earth. And marine microbes remove so much carbon dioxide from the atmosphere that some scientists see them as a potential solution to global warming.

Yet even as scientists describe seemingly endless variations on the cosmopolitan microbial lifestyle, the concept of a bacterial species remains elusive. Some bacterial species (such as anthrax) appear to have little genetic variation while in others (such as Escherichia coli) individuals can have completely different sets of genes, challenging scientists to explain the observed diversity.

The emerging field of environmental genomics (or metagenomics) aims to capture the full measure of microbial diversity by trading the lens of the microscope (and biochemistry) for the lens of genomics (and bioinformatics). By recovering communities of microbial genes where they live, environmental genomics avoids the need to culture uncooperative organisms. And by linking these data to details relating to sequence collection sites, such as pH, salinity, and water temperature, it sheds light on the biological processes encoded in the genes.

The largest metagenomic dataset collected so far comes from the *Sorcerer II* expedition, named after the yacht J. Craig Venter transformed into a marine research vessel. In a pilot study of the Sargasso Sea, Venter's team identified 1.2 million genes and inferred the presence of at least 1,800 bacterial species. But the genetic and taxonomic diversity of the data imposed new challenges on existing genome assembly methods and other analysis techniques. The researchers designed the *Sorcerer II* Global Ocean Sampling (GOS) expedition to see if collecting more samples would improve their assembly and lead to a better estimate of the number and diversity of microbial genes in the oceans.

And now, in three new studies, Venter's team has combined the expedition's latest bounty—6.5 million sequencing “reads”—with the Sargasso Sea data. The result is a geographically diverse environmental genomic dataset of 6.3 billion base pairs—twice the size of the human genome. (To learn about the voyage and sampling methods, see Box 1.) In the first paper, Douglas Rusch, Aaron Halpern, and colleagues attempt to describe the immense amount of microbial diversity in the seas, and determine how—or if—that diversity is structured and what might be shaping that structure. In the second paper, Shibu Yooseph et al. study the millions of proteins in the GOS sequences to see if we're close to discovering all the proteins in nature. And in the third study, Natarajan Kannan, Gerard Manning, and colleagues classify thousands of kinases into 20 distinct families, revealing their structural and functional diversity and an unexpected importance in prokaryotic regulation.

**Box 1. Following the *Sorcerer II*'s Hunt for Microbes**

The *Sorcerer II* expedition was inspired by the British *Challenger* expedition (1872–1876), a pioneering oceanography research project that discovered hundreds of new genera and nearly 5,000 new marine species. Its gun stations replaced with research stations, the *Challenger* circumnavigated the oceans, stopping every 320 kilometers to recover specimens from bottom, intermediate, and surface depths to explore the diversity of macroscopic marine life. At each stop, the crew recorded the location, what they used to extract the sample, the depth of the sample, and several observations related to water and atmospheric conditions. The *Sorcerer II* followed a similar sampling schedule, traveling nearly 9,000 kilometers to collect samples of microbial marine life and record the water's location, depth, pH, salinity, and temperature.

**Figure:**
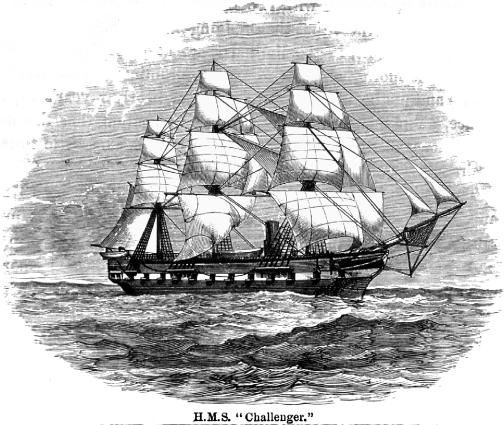
H.M.S. *Challenger* (Image: NOAA, Steve Nicklas)

The GOS crew collected samples from surface waters of diverse, mostly marine aquatic environments. The samples were collected between August 2003 and May 2004 during a six-leg journey that followed a path from northeastern Canada to the South Pacific Gyre. Venter's crew collected microbial samples by pumping 200 liters of surface seawater through a series of increasingly fine filters, which they labeled, froze, and sent back to the lab of the J. Craig Venter Institute in Maryland.

After a stop in the Gulf of Maine, the expedition sampled three sites along Nova Scotia, including a “highly eutrophic” coastal embayment in Halifax. The crew set sail again in November, starting in Newport Harbor, Rhode Island, and ending in the Delaware Bay, one of several estuaries targeted on the journey.

The next leg began in Chesapeake Bay. The largest US estuary, Chesapeake Bay contains a rich mix of freshwater and marine organisms. Estuaries are complex hydrodynamic environments that are highly sensitive to runoff from agricultural and urban development (which can dump massive amounts of nitrogen and phosphorous into watersheds). Microbial communities collected from estuaries promise to provide valuable insights into the metabolic and physiological adaptations required by such environments. Continuing down the Atlantic seaboard, the expedition stopped near Cape Hatteras, North Carolina, and the Florida Keys before passing through the Caribbean and ending near Panama, where the crew collaborated with scientists at the Smithsonian Tropical Research Institute.

The fourth leg of the voyage sampled sites in the Eastern Pacific, including Cocos Island, about 500 kilometers southwest of Costa Rica. A highly productive ecosystem inhabits the waters off the island, a result of ocean currents buffeting the coast and causing nutrient upwellings that mix with warm surface waters. The crew made one last stop in the open ocean, then headed for the Galapagos Islands.

Owing in part to its position near major ocean currents and atmospheric transition zones, the Galapagos Archipelago sits within a hydrographically complex region. Unique oceanographic features there support a diverse set of habitats and endemic species, found within several discrete zones distinguished by temperature. This microbial mother lode held the crew's attention for two months, while they extensively sampled the region.

By early March 2004, the crew had collected the last three samples used in these studies, from two open ocean sites and a lagoon in a coral reef in the South Pacific Gyre. Follow these links to learn more about the *Sorcerer II* (http://www.sorcerer2expedition.org/version1/HTML/main.htm) and the *Challenger* (http://hercules.kgs.ku.edu/hexacoral/expedition/challenger_1872-1876/challenger.html) expeditions.

**Figure:**
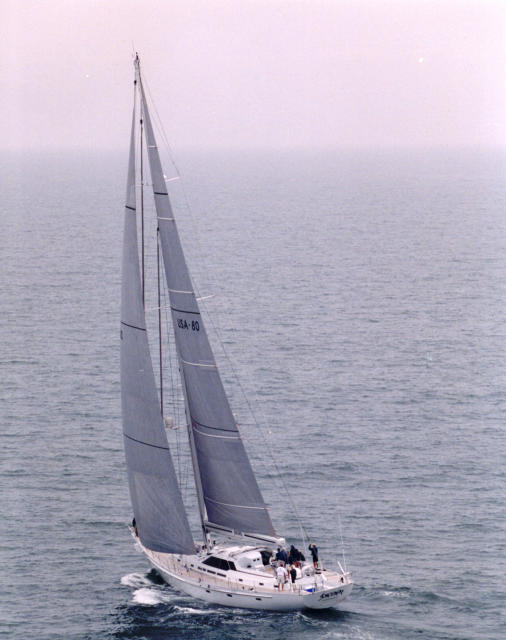
The *Sorcerer II* (Image: J. Craig Venter Institute)

## Extracting Meaning from Metagenomic Datasets

The GOS samples used in the Rusch et al. study were collected over the course of a year from a wide range of aquatic environments—including estuaries, lakes, and open oceans—then pumped through serial filters. After extracting the genetic material from the microbe-encrusted filters, Rusch et al. used shotgun sequencing to study the genes present in the samples. DNA is forced through a tiny nozzle that smashes it into bits; the fragments are cloned and the letters of the genetic code are scanned from both ends to create “reads.” Reads are then assembled, much like a jigsaw puzzle, starting with contiguous fragments (“contigs”) that are then mapped onto “scaffolds,” which order and orient sets of contigs on a chromosome. (For more on shotgun sequencing, see Box 2.) Using a conservative sequence similarity requirement, most reads failed to assemble, suggesting that the samples contained great microbial diversity.

**Box 2. Bioinformatic Methods at a Glance**

Bioinformatics relies on statistics and computer power to synthesize and interpret huge datasets. Here's a brief introduction to some of the environmental genomics methods used in the GOS studies.

**Shotgun sequencing** decodes genetic material by randomly shredding it into millions of fragments. The DNA sequence of each end of a fragment is determined; the two ends of a given fragment (or insert) can be associated, and constitute a “mate pair.” These random sequencing “reads” are then reassembled with a computer. Based on sequence similarity, overlapping reads are identified and merged into longer sequences called “contigs.” Contigs are organized into larger (but not necessarily continuous) pieces of a genome, called “scaffolds,” based on mate pairs. The resulting assemblies can link genes to their regulatory elements, guide investigations of biological pathways, and connect unknown sequences with taxonomic markers to suggest evolutionary relationships.

**Sequence similarity detection** allows functional and taxonomic characterization of genomic sequences. Once the shotgunned sequences have been organized into a library of sequence “scaffolds” and translated into hypothetical proteins, the next step uses sequence similarity to figure out what the proteins are and to identify families. Similarity can also associate a new sequence with an approximate location on the tree of life.

**Sequence–sequence (pairwise) methods,** the first step for identifying closely related sequences, compare all sequences to all other sequences in a pairwise manner. These methods (such as BLAST) allow all collected sequences to be compared with one another (and with all sequences already available in public databases) and reliably clustered into families of related sequences with high sequence similarity, or homology.

**Profile methods** are used to identify more remote relationships. Profile methods use multiple sequence alignments of previously identified families to compute “position-specific scoring matrixes” (PSSMs). Each position in the alignment is associated with a set of scores that reward or penalize the alignment of a given amino acid to the position. Profile methods can be more sensitive than simple sequence similarity methods because they give more weight to signals at sites that are conserved within a protein family and less weight to more variable positions.

Initially, the advantages of profile methods for detecting remote homology were limited to well-characterized families, as construction of a profile required some expertise. However, this changed with the fully automated integration of this step into PSI-BLAST. PSI-BLAST begins with a pairwise (sequence–sequence) similarity search, but then iteratively runs alternating steps of building a profile from the current set of similar sequences and using the profile to re-search the database for additional matching sequences.

**Hidden Markov models** (HMMs) employ statistical methods to model the likelihood of different amino acids at any given position of the sequence in an underlying alignment. Like some profile methods, HMMs use a probability-based method to determine the score of aligning an observed amino acid to a given position in a protein family, but HMMs improve upon profiles by more sophisticated modeling of variation in protein length, storing the probabilities of insertions or deletions at each position of the model. HMMs have a good track record for identifying more distantly related protein sequences.

**Profile–profile methods** are the most recent enhancement to sequence homology detection methods. As the name suggests, profile–profile methods compare one profile to another. Because each profile implicitly encodes more information than a single sequence, these methods identify relationships that cannot be detected by comparing individual sequences.

With only a bare bones assembly to guide their investigation, Rusch et al. tried a different approach. They used the 584 completed and draft microbial genomes already available in public databases as points of reference and relaxed search parameters to detect even remote similarity to GOS sequences. Although the majority of GOS reads matched up with one or more of the reference genomes, the loose criteria prevented the researchers from drawing meaningful inferences about kinship.

To boost their inference power, they required that similarity to a reference genome extend nearly the full length of a read (producing “recruited reads”). A substantial majority of reads failed this criterion, with only 30% of the GOS data being recruited. The bulk of these aligned to three genera of widely distributed marine microbes—Pelagibacter, Synechococcus, and Prochlorococcus—which accounted for about 15% of the recruited reads. The remaining recruited reads appear to signal conserved genes rather than closely related organisms. Most of the GOS sequences failed to be identified, in part because so few surface water microbes have been sequenced.

### A novel comparative genomic method

Focusing on the reads that recruited to these most abundant genera, Rusch et al. generated “fragment recruitment plots.” These graphics represent relatedness and diversity of environmental sequences to a reference genome by showing where a read aligns with the reference genome (indicated by a horizontal bar) and its degree of similarity to the reference sequence (indicated by its vertical position). Recruited reads were color-coded based on sample origin to indirectly depict their associated metadata (for example, salinity and pH). These plots provided a visual tool to explore genetic diversity at the sequence and gene level, genome structure and evolution, and taxonomic and evolutionary relationships. (For more on fragment recruitment plots, see the accompanying poster, doi:10.1371/journal.pbio.0050077.sd001.)

Distinct recruitment patterns, easily detected by bands of color, emerged for each organism. In some cases, a single reference genome had multiple color bands, distinguished by their similarity and sample provenance. Because bands appeared to represent unique, closely related, and geographically distinct populations—and showed a novel level of diversity across the entire genome—the researchers termed each band a subtype. A tremendous amount of sequence diversity appeared in the subtypes, which also harbored substantial sequence variation at the protein level, some likely reflecting adaptations to local environments. This finding reveals a potential locus of microbial diversity—at the level of subtype rather than at the level of species, or ribotype (based on a segment of a ribosomal RNA gene called 16S rRNA)—and offers clues to why it emerged (perhaps in response to local pressures) and how it evolved.

### A novel sequence assembly method

Because such high levels of sequence diversity among organisms confound standard whole genome assembly software, and most of the GOS data correspond to organisms for which there is no appropriate reference genome, Rusch et al. used an “extreme assembly” approach to investigate the genomes of other abundant GOS populations. They used greatly reduced requirements for sequence similarity in the assemblers to generate longer contigs and capture more of the GOS data in an assembly. While some of the resulting larger assemblies corresponded to known reference genomes, others did not, allowing the researchers to study microbes without cultivated or sequenced counterparts. And because these larger assemblies could potentially provide functional insights into uncharacterized organisms, they might identify conditions that would allow scientists to grow them in the lab.

Many of the large contigs failed to align in any significant way with known genomes, so the researchers tried to match them with “seed fragments” from known taxonomic groups. By starting assembly from reads mated to the 16S rRNA gene—one of the most common marker genes used for classifying microbes—they could generate large contigs associated with many of the abundant GOS ribotypes. Fragment recruitment plots of these assemblies again revealed multiple subtypes, providing further support for the presence of multiple evolutionarily distinct subtypes within a given ribotype.

### Evidence for environmental adaptations

A computational approach designed to identify groups of samples with similar genomic content revealed that tropical and temperate samples shared the least amount of genomic material. Some samples, however, were very similar.

While untangling all the factors that may affect genetic makeup of a sample is beyond current datasets and methods, the researchers demonstrated that specific genetic differences can be related to environmental factors. Several genes occurred up to seven times more frequently in a pair of samples from the Caribbean than they did in a pair from the eastern Pacific, even though both pairs had similar ribotype and genetic profiles. Many of these genes govern the metabolism and transport of phosphate (required for microbial growth), likely reflecting functional adaptations in the microbial communities to the measured differences in phosphate availability in the Caribbean and Pacific samples.

The researchers also explored diversity at the gene level by looking for evidence of functional differences in one gene family, proteorhodopsins, light-activated proton pumps with a slightly murky biological role. Proteorhodopsins were abundant in all the GOS and Sargasso Sea samples. In keeping with the diverse light environments sampled during the expedition, the researchers found a strong correlation between sequence variation and sample provenance. They hypothesize that the distribution of given variants reflects adaptation to the most abundant light spectra in their habitats.

Altogether, these results reveal the power of metagenomic approaches to capture the true measure of microbial diversity by uncovering genomic differences that would not have been apparent using traditional marker-based approaches. The breadth of this newly revealed diversity may come as a surprise to even inveterate microbe hunters.

## The Expanding Protein Universe

Along with insights into microbial diversity, metagenomics promises to help us understand the vast number of proteins in nature. By randomly sampling DNA sequences from communities of organisms, metagenomic studies overcome selection and culturing biases that arise from focusing on a particular organism or a set of proteins, to provide an expansive view of protein diversity and evolution.

Proteins are typically grouped into families based on their evolutionary relationship, which can then be used to guide investigations of their biological roles. Proteins in the same family share similar amino acid sequences and three-dimensional conformations. Using amino acid sequence similarity as a measure to identify and group protein sequences from the GOS data with sequences from a comprehensive set of known proteins, Shibu Yooseph et al. evaluated the impact of the GOS data on our understanding of known proteins and studied the rate of discovery of protein families with new sequences. To group related sequences and predict proteins, they developed a novel sequence clustering technique based on full-length sequence similarity.

### Identifying proteins in metagenomics data

Hypothetical proteins can be predicted by searching for open reading frames (ORFs), sequences flanked by nucleotide triplets (called codons) that signal the beginning and end of translation but don't necessarily encode a protein. Because the GOS data contain many fragmentary sequences, Yooseph et al. allowed ORFs to be terminated at the end of a sequence, resulting in a partial or truncated ORF. They used the ORFs to generate a set of predicted proteins based on the results of a series of clustering steps and statistical analyses.

After performing pairwise comparisons (of every sequence against every other sequence) of the more than 28 million sequences in the combined dataset, the researchers identified conserved groups of sequences after accounting for redundancy due to identical and near-identical sequences. They then used profile methods to merge and expand these groups of sequences. While pairwise comparisons capture the most closely related sequences (or homologs), profile methods (the researchers used both PSI-BLAST and hidden Markov models) detect more distantly related sequences by combining homologs into multiple sequence alignments to generate “profiles.” (For more on these methods, see Box 2.)

From the clusters obtained by the above procedure, clusters of spurious sequences (that overlap true protein regions on the genome) were identified in addition to clusters of noncoding conserved sequences (based on tests showing no selection on their codons). Sequences in these clusters were removed; those remaining were labeled as predicted proteins. The researchers identified nearly 6 million proteins in the GOS dataset—1.8 times the number already in public databases. Comparing the predicted protein clusters to known prokaryotic and nonprokaryotic protein databases revealed GOS counterparts in nearly all known prokaryotic protein families; nearly 2,000 clusters appeared unique to the GOS dataset.

Since they couldn't use sequence similarity to infer function for the unique GOS sequences, the researchers relied on the assumption that proteins with similar roles are more likely to reside in the same genomic neighborhood. This analysis implicated several GOS-only clusters in photosynthesis or electron transport. Such clusters may come from viruses, as many viral parasites of photosynthetic bacteria express the photosynthetic genes of their hosts. Interestingly, though most of the sequences in GOS-only clusters appeared to be bacterial, a higher than expected proportion of them were flagged as viral. If such novel GOS protein families pan out as viral, the researchers argue, “we are far from exploring the molecular diversity of viruses.”

### Insights into evolutionary and functional diversity

To compare ocean versus terrestrial life at the biochemical level, Yooseph et al. compared GOS sequences to those of land-dwelling prokaryotes. Nearly 70% of protein domains varied between the two classes of microbes, mostly reflecting the distinct biochemical requirements of the two environments, as well as the different taxonomic groupings in the two datasets. The researchers were surprised to find little evidence of domains specific to gram-positive bacteria (defined by their unique cell wall), even though this bacterial group makes up nearly 12% of the GOS dataset. They also found a relative dearth of components related to flagella (the whip-like tail of microbial motility), possibly reflecting the reduced need for self-propulsion in the ocean.

Using a comprehensive protein family database (called Pfam), the researchers compared the kingdom distribution of known protein domains in the GOS data to that of proteins in public databases. In this process, some families that were previously thought to be single-kingdom turned out to have members in multiple kingdoms. For example, indoleamine 2,3-dioxygenase (IDO), an enzyme linked to the immune system in mammals, was considered unique to eukaryotes. But the IDO Pfam search turned up matches to ten GOS sequences identified as bacterial—suggesting that the proteins may have arisen much earlier than previously thought, or perhaps arose through lateral gene transfer (from an unrelated organism).

The sheer size of the GOS dataset—which nearly doubles the number of proteins—greatly expands the functional diversity of known protein families, providing valuable insights into their evolution. For example, the researchers found a 10-fold increase in the number and type of proteins involved in repairing ultraviolet radiation damage, likely reflecting the hazards of living in surface waters. A similar boost in phosphatases—which function in such fundamental biological processes as cell signaling, development, and cell division—highlighted important differences in the way one phosphatase (protein phosphatase 2C) functions in prokaryotes and eukaryotes.

And the unexpected abundance of a nitrogen metabolism catalyst typically associated with eukaryotes (type II glutamine synthetase) suggested two possible evolutionary mechanisms: either lateral gene transfer from eukaryotes, or gene duplication prior to the divergence of prokaryotes and eukaryotes. (The researchers suspect gene duplication.) The diversity of the GOS sequences also promises to characterize sequences with no similarity to known sequences (known as ORFans): over 6,000 ORFans pair up with GOS sequences representing some 600 organisms, paving the way for further study of their identity and function.

As GOS protein predictions are tested, some of these proteins will expand existing protein families while others will carve out GOS-specific families. Both results will help researchers determine priority targets for structural studies—an essential strategy for dealing with the flood of protein discoveries. And given that the GOS sequences represent mostly microbes from the ocean's surface—yet point to substantial viral diversity as well—the rate of protein discovery indicates that a comprehensive catalog of proteins in nature is far from complete.

## Variations on a Theme: A Single Fold Spawns a Diverse Kinase Superfamily

Cellular life chugs along under the power of enzymes, proteins that catalyze the scores of chemical reactions required for life. One of the largest protein families in eukaryotes, the eukaryotic protein kinases (ePKs), regulates the activity of a large fraction of all proteins and almost all biological pathways by phosphorylating proteins. Phosphorylation activates its target by transferring a phosphate group from adenosine triphosphate (ATP) to a specific amino acid on the protein, releasing energy and inducing structural changes that alter the protein's activity. (Dephosphorylation removes the phosphate group, restoring the protein to its original conformation and inactive state.) One cell can contain hundreds of different protein kinases, each charged with phosphorylating one or many different proteins.

Bacteria and other prokaryotes, conventional wisdom held, rely mostly on structurally distinct kinases (histidine kinases) to mediate protein phosphorylation and cell signaling. But it now emerges that ePK-like kinases (ELKs), once thought to be minor players, are more prevalent and widespread than the histidine kinases. Although ePKs and ELKs typically exhibit very low sequence similarity, they share similar phosphorylation mechanisms and the same structural fold (the protein kinase–like, or PKL, fold).

Since PKL kinases conserve both fold and mechanism of action, they provide a robust model for determining how sequence variation corresponds to functional diversity. Unfortunately, comprehensive comparisons had been frustrated by a lack of sequence information for the prokaryotic ELK families relative to the well-studied eukaryotic domains. But now, thanks to the *Sorcerer II* expedition, sequence databases are brimming with microbial sequences, including a 3-fold increase in ELK sequences. Taking advantage of the bounty, Natarajan Kannan, Gerard Manning, and colleagues surveyed the global PKL landscape, and identified over 45,000 PKLs, which they classified into 20 families. Surprisingly, PKLs appear to usurp the histidine kinases as the core regulator of prokaryotic signaling and cell behavior.

### Cataloging the number and diversity of PKL families

To detect kinase sequences, Kannan et al. searched over 17 million predicted proteins in the GOS dataset and 5 million-plus predicted and known protein sequences in public databases. Kinase sequences were detected using hidden Markov model (HMM) profiles of known PKLs along with a model that predicts kinases on the basis of a few ultra-conserved motifs. The sensitivity of the HMMs allowed the researchers to discover very remote new members of these families and to classify and organize the tens of thousands of sequences. Both approaches iterate through multiple runs of the clustered results to refine the family alignments and to classify clusters with little similarity to known PKL families as potentially novel. (For more on these methods, see Box 2.)

The public databases, it turned out, harbored nearly 25,000 ePKs and over 5,000 ELKs. Over 16,000 GOS sequences fell into 20 PKL families—doubling the size of most families. Three main superfamily clusters emerged, distinguished by the most abundant members: choline and aminoglycoside kinases (CAKs), a “particularly diverse” family harboring kinases that facilitate colonization by beneficial and pathogenic bacteria; ePKs, almost exclusively eukaryotic except for a similar bacterial kinase (pknB); and a cluster of kinases, including Rio and Bud32, that are conserved between archaea and eukaryotes. Three families bore no sequence similarity to any other families save for a group of key motifs.

Overall, the 20 families exhibit significant functional and sequence diversity. Most of the families have not yet been fully investigated, though they do include some characterized members. Those with known kinase activity target small molecules (such as lipids and amino acids) and seem to play regulatory roles, in contrast to many other structurally unrelated small molecule kinases, which affect metabolism.

### Functional diversity springs from a set of core residues

Because sequence similarity ranged from “very low to almost undetectable,” the researchers used sequence profiles—models built from entire families to highlight their core characteristics—to both discover and classify kinase sequences. They found several novel families, and greatly extended the breadth of previously defined families. With these methods to refine the relationships within and between PKL families, the researchers explored the traits that unite or distinguish them.

Ten key amino acid residues of the catalytic domain consistently turned up in each family. This “core pattern of conservation,” the researchers explain, represents an ancient evolutionary innovation, spanning not just the three divisions of life—which diverged 1–2 billion years ago—but also the diverse families. The conservation of these residues across and within the families suggests that they play an essential role. And, indeed, six of those already characterized mediate ATP binding and catalysis.

Yet despite the seemingly universal presence of the ten residues, their occurrence in individual subfamilies showed a surprising pattern: all but one of these “core” residues had either disappeared or changed in individual families—though the proteins retained their fold and function—suggesting an unexpected flexibility for catalytic cores. To test this possibility, the researchers focused on one of the ten residues—the catalytic lysine K72, which repositions ATP's phosphates. Present in ePKs, K72 is replaced by a different conserved amino acid in three CAK subfamilies. These subfamilies had corresponding substitutions near other key motifs, and structural modeling showed how these coordinated replacements could still result in an active enzyme.

A number of features (including amino acid motifs and secondary structure) emerged as family-specific, being highly conserved within but not between families. And as was seen in the CAK analysis, many family-specific residues occur near one of the ten key residues, suggesting that they may help direct substrates to the catalytic core or influence the nature of the reaction.

### Evolutionary insights and beyond

Altogether these results reveal the vast functional and phylogenetic diversity that can occur in even just a subset of proteins, even though they retain a common catalytic fold and function. The massive sequence comparisons in this study not only identified the core of the PKL kinase, but also revealed the specific motifs underlying each family, including the ePKs. And the flexibility of several key regions within ePKs may underlie the huge expansion of these enzymes in eukaryotes. This structural flexibility may give kinases the ability to integrate multiple regulatory signals, and account for their almost universal involvement in the regulation of eukaryotic pathways.

These results set the stage for more in-depth structural and biochemical studies to elucidate the diverse functions carried out by these critical regulators of cell behavior. This study also demonstrates how metagenomic datasets, by covering an unbiased diversity of life, can refine our understanding of well-studied protein families, such as the ePKs, and shed light on their evolution. Kannan et al. hope that others take advantage of the environmental metagenomic largesse to pursue “similar insights into virtually every gene family with prokaryotic relatives.”

## 

